# The orthopterans of the rice agroecosystem in western Lomellina (Lombardy, Italy)

**DOI:** 10.3897/BDJ.6.e24203

**Published:** 2018-05-02

**Authors:** Davide Giuliano, Giuseppe Bogliani

**Affiliations:** 1 Department of Earth and Environmental Science, University of Pavia, Pavia, Italy

**Keywords:** Orthoptera, check-list, rice fields, dispersal capacity, habitat specificity, farming practices, northern Italy

## Abstract

Rice fields represent a valuable surrogate habitat for many wetland species, playing an important role for biodiversity conservation in human-managed landscapes. Despite the fact that several taxonomic groups have been thoroughly investigated in this agroecosystem, little is known about the orthopteran fauna which lives in and around rice paddies, especially in Europe. In this paper, we provide a first description of the orthopteran assemblages hosted in the rice agroecosystems of northern Italy, trying to evaluate their conservation value through an analysis of species ecological traits (habitat specificity and dispersal capacity). During field samplings in summer 2016, we detected 25 orthopteran species. The 24% of the community was composed by habitat specialist species and the 56% of the sampled *taxa* was characterised by high dispersal capacities. Rice fields are an extremely dynamic ecosystem, characterised by the continuous succession of flooding and drying periods and conditioned by many other farming activities. Consequently, the orthopteran fauna in rice crops is mainly composed of species well adapted to sudden environmental changes. On the other hand, rice fields represent a particular biotope, providing a suitable habitat especially for hygrophilous species, which are otherwise restricted to scattered marsh areas. In order to preserve orthopteran diversity in rice agroecosystems, sustainable farming practices should be applied, especially by preserving and restoring marginal semi-natural habitats, by reducing grass management intensity on paddy banks and by discouraging rice cultivation in dry soils.

## Introduction

Orthopterans (Insecta: Orthoptera) are often considered as key components of farmland biodiversity. Indeed, these insects may provide several ecosystem services in arable lands, e.g. limiting weed expansion through seed predation (Ichihara et al. 2014) and supplying considerable food resources for birds (Barker 2004, Rodríguez and Bustamante 2008, Bretagnolle et al. 2010, Fasola and Cardarelli 2014). In addition, grasshoppers proved to be good indicators of grassland ecosystems health, as they strongly respond to management intensification, both by decreasing species richness and population density (van Wingerden et al. 1992, Báldi and Kisbenedek 1997, Fischer et al. 1997, Marini et al. 2007).

In Europe, the highest orthopteran species richness is usually found in open habitats, such as grasslands, heathlands or Mediterranean shrublands (Hochkirch et al. 2016), thus suggesting the potential role of traditional agricultural landscapes in maintaining a high diversity of these insects. Unfortunately, in the last decades, agriculture underwent a widespread intensification in this geographical area, increasing landscape homogenisation and reducing the availability of semi-natural habitats (Benton et al. 2003, Tscharntke et al. 2005). This type of anthropogenic pressure is considered one of the main causes for orthopterans’ decline in Europe, since many species are now threatened by the loss of suitable habitats (Hochkirch et al. 2016). Therefore, monitoring programmes to assess the species status and distribution in farmlands are needed, especially in order to identify effective management strategies for their conservation.

Rice fields are an agricultural landscape particularly extended in the western portion of the Po Plain (northern Italy). Similarly to other crops, rice cultivation recently underwent a pronounced intensification in this area (Bogliani 2008), losing most of the biodiversity conservation value previously ascribed to this agroecosystem (Fasola and Ruiz 1996, Lawler 2001, Katayama et al. 2015).

Unlike other taxonomic groups (e.g. birds, amphibians, aquatic insects etc.), little is known about the orthopteran fauna which lives in rice paddies. Indeed, only few studies have been carried out on this subject, mainly in Asian countries (Chitra et al. 2000, Ghahari et al. 2009), where many species are considered as potential rice pests. Concerning Italy, a specific research on the orthopterans in rice crops is currently lacking. To date, faunistic data about this habitat could only be inferred from the existing regional (Sindaco et al. 2012) or national (Massa et al. 2012) checklists, often without any detailed information on species occurrence in rice paddies.

This paper aims to provide a first description of the orthopteran fauna hosted in the rice agroecosystems of the western Po Plain, providing further important data to assess the value of rice crops for biodiversity conservation.

## Materials and methods

### Study area

**Figure 1.**
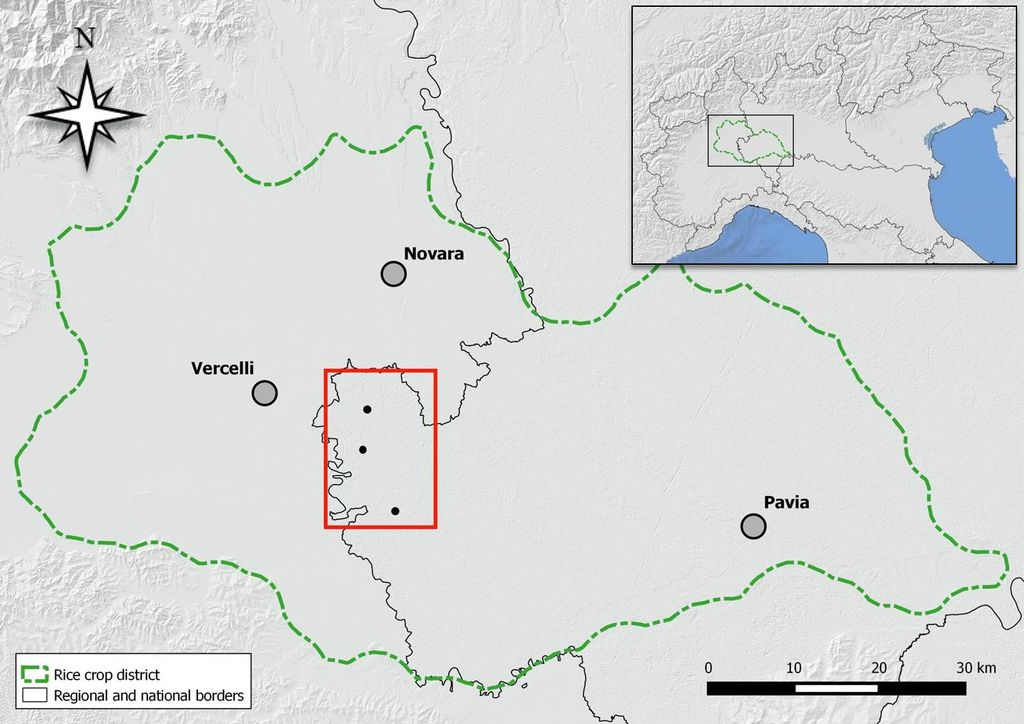
Map of the whole rice crop district in northwestern Italy, enclosed by the green dashed line. The three black dots within the red square (western Lomellina) identify the investigated sites in this study.

The study was carried out in three sites within the rice crop district of western Lomellina (Lombardy, northern Italy), located in the municipalities of Robbio (45°18’45.02” N; 8°35’18.53” E), Rosasco (45°16’10.59” N; 8°34’51.07” E) and Zeme (45°11’42.55” N; 8°38’25.01” E). The overall sampling area was included in a north-south range of 20 km, within a landscape heavily dominated by rice fields (Fig. 1).

In order to provide a specific characterisation of the orthopteran fauna in rice crops, the data reported in this paper were collected only in habitat structures typical of this agroecosystem, namely paddies, field banks, irrigation canals and nearby unpaved country roads. Other landscape components, such as wooded patches, hedgerows and tree lines, were not investigated during this study.

### Data collection

This research is part of a biodiversity monitoring programme (“Rice for Life” project) carried out in 2016 in western Lomellina in order to evaluate the effects of different agricultural techniques on a wide range of taxonomic groups in rice agroecosystems. In this framework, orthopterans were used as model organisms to assess the impacts on insect diversity caused by grass management on rice field banks (Giuliano et al., *under review*).

In accordance with the monitoring project requirements, orthopterans were mainly collected on paddy levees, following a standardised sampling method. Therefore, these insects were sampled monthly, in the period between June and September, by means of a visual census in 50 sampling plots, each one made up by a standard surface of 1x10 m.

However, a consistent amount of the data presented in this paper has also been collected outside the standardised sampling design. Indeed, between May and September 2016, orthopterans were randomly sampled in rice crops, investigating in particular those habitats not considered in the monitoring project previously described (e.g. paddies, irrigation canals and farm roads). These additional samplings were performed in order to ensure the compilation of a species list as complete as possible for the whole rice agroecosystem.

In both cases, orthopterans were collected manually and most of the individuals were identified directly in the field, while those not readily identifiable were suppressed in a 70% ethanol solution and recognised later in laboratory. Species identification was carried out mainly by analysing the morphological traits, following the keys of Coray and Thorens (2001), Baur et al. (2006) and Massa et al. (2012). All the collected individuals were dried and included in a reference collection (now preserved at the Department of Earth and Environmental Sciences, University of Pavia).

Moreover, during field surveys, several individuals were also identified by listening to their acoustic signals, comparing them with the recordings provided by Odé and Fontana (2002) and Massa et al. (2012). This technique allowed the detection of species otherwise difficult to observe and being particularly effective for those characterised by an unmistakable song.

### Dispersal capacity and habitat specificity

In order to assess the conservation value of the orthopteran assemblages observed in the rice agroecosystem, we collected information about the ecological traits of all the sampled species, focusing in particular on their dispersal capacity and habitat specificity. These features are considered important factors in determining species sensitivity to habitat loss and human disturbance, with sedentary and habitat specialist *taxa* often more susceptible to local extinction events (Reinhardt et al. 2005).

Dispersal capacity was measured using the Mobility Index developed by Reinhardt et al. (2005) and afterwards applied by Marini et al. (2010). Each species was classified into one of three broad mobility classes: sedentary, intermediate dispersers and mobile species. All apterous and brachypterous orthopterans were classified as sedentary, while readily flying species were assigned as mobile. Furthermore, for species with wing dimorphism, we considered the most common form.

Concerning habitat specificity, each species was assessed according to its moisture preferences, following the procedure reported by Reinhardt et al. (2005). As for dispersal capacity, we assigned orthopteran species to one of three broad classes: habitat specialists, medium specialised species and generalists. Xerothermophilous and hygrophilous species were classified as habitat specialists, while orthopterans with broad ecological requirements were considered as generalists.

The species not treated by Reinhardt et al. (2005) and Marini et al. (2010) were assigned to mobility and habitat specificity classes according to their wing development and their habitat requirements, gathering this information from Baur et al. (2006) and Massa et al. (2012).

## Results

During the study period, we collected 348 individuals, belonging to 25 species (10 Ensifera and 15 Caelifera) (Table 1). Due to their identification complexity, mainly related to the analysis of the chromosomal makeup (Massa et al. 2012), the individuals ascribed to the genus *Gryllotalpa* were identified only as a species complex.

Rice field banks hosted the higher species richness between the investigated habitats (17), while in farm roads (10), irrigation canals (8) and rice paddies (6), the observed number of species was lower. Nevertheless, these results should be interpreted carefully, since our sampling effort has been higher on paddy levees and not standardised between habitats.

**Figure 2.**
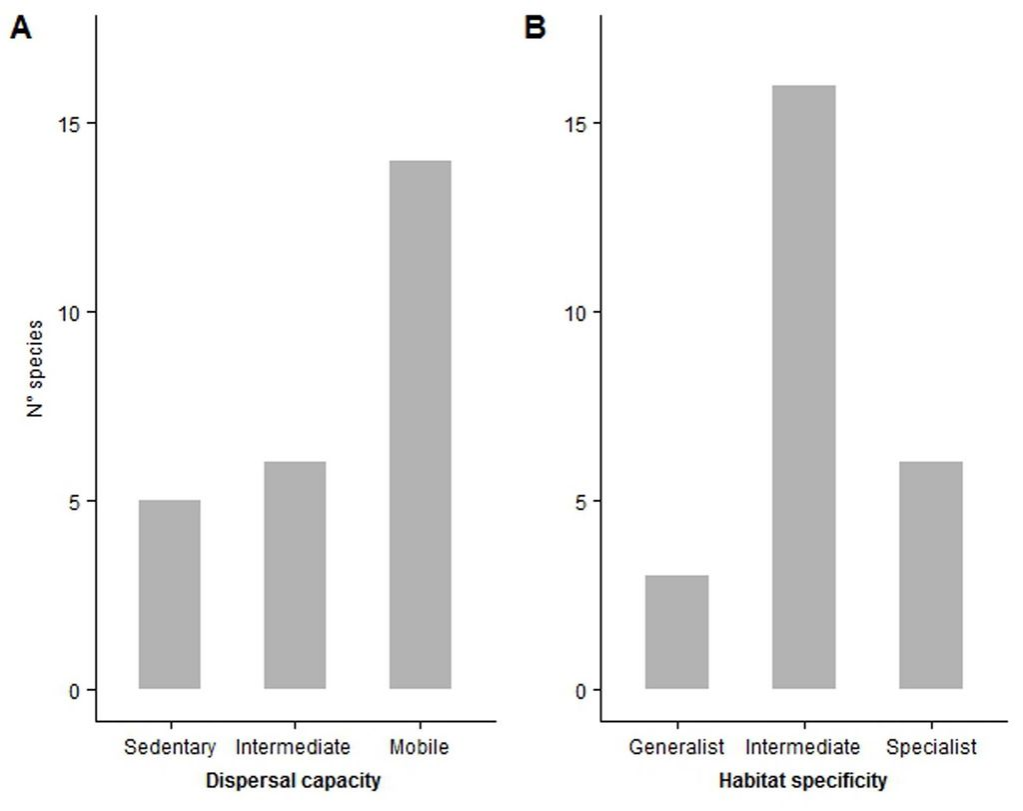
Barplots which report the number of species belonging to each dispersal capacity (A) and habitat specificity (B) class in the orthopteran assemblage observed in the rice agroecosystems of western Lomellina.

Concerning ecological traits, the orthopteran assemblage was dominated by highly mobile species (14), while intermediate dispersers (6) and sedentary species (5) were little represented in the rice agroecosystem (Fig. 2a). Medium specialised species represent a large proportion of the community observed in rice crops (16), while habitat specialist (6) and generalist (3) orthopterans were less common in the investigated habitats (Fig. 2b).

According to the IUCN European Red List of Orthopterans (Hochkirch et al. 2016), most of the species observed in the rice agroecosystem are considered of least conservation concern. However, it is important to remark on the presence in rice crops of *Paracinema tricolor*, assessed as *Near Threatened* in Europe.

## Discussion

To date, the most detailed information about the orthopteran fauna in the western Po Plain were reported by Sindaco et al. (2012). These authors provided a list of 62 species for the eastern Piedmont lowlands, a geographical sector very close to our study area, also characterised by extended rice crops. According to this publication, the 25 species sampled in the rice agroecosystem of western Lomellina represent more than one third (40.3%) of the orthopterans which likely occur in this portion of the Po Plain. Hence, rice fields seem to play an important role in maintaining orthopteran diversity in northern Italy lowlands, especially because the data reported by Sindaco et al. (2012) are cumulative for a wide range of habitats, including many natural sites. Moreover, the occurrence of a considerable proportion of specialised species (24.0%) in rice crops further underlines the environmental value of this agroecosystem for these insects.

Rice fields represent a particular biotope, typically characterised by a temporary aquatic ecosystem (Fasola and Ruiz 1996): paddies are managed through a cycle of flooding and drying periods according to husbandry requirements, with the almost permanent presence of water during summer. Therefore, rice crops are particularly suitable for orthopteran species with specific moisture requirements, especially providing an appropriate habitat for hygrophilous *taxa*, which are otherwise restricted to scattered marsh areas. For this reason, rice fields could constitute a valuable habitat for the conservation of these species, since the widespread deterioration of natural wetlands, which occurred in the last century, is considered one of the major threats for hygrophilous orthopterans in Europe (Hochkirch et al. 2016).

This is the case of *Paracinema tricolor*, which is strongly related to wetland habitats and included in the *Near Threatened* category by Hochkirch et al. (2016). The populations of this species recently became severely fragmented in Europe, being particularly affected by detrimental drainage activities and by land use intensification. Nevertheless, *P. tricolor* is particularly abundant in flooded rice paddies, suggesting the effectiveness of this type of agroecosystem as a surrogate habitat for the conservation of this species.

Although rice crops have been proved to be a suitable habitat for hygrophilous orthopterans, during our research we also noticed the occurrence of several thermophilous species. Landscape structures, such as bare banks, mown levees and unpaved country roads, often provide unexpected xerothermic micro-habitats in rice fields, improving the suitability of this agroecosystem also for species adapted to dry and warm conditions. This result highlights the value of habitat heterogeneity in maintaining a high orthopteran diversity in rice crops, especially allowing the coexistence in the same ecosystem of species with different ecological requirements.

Despite the described environmental value of rice agroecosystems for orthopteran diversity, a large proportion of the species sampled in our study area is characterised by high dispersal capacities (56.0%), a typical feature of arthropods adapted to temporary and highly disturbed habitats (Ribera et al. 2001, Dziock et al. 2011). As previously mentioned, rice fields are an extremely dynamic ecosystem, characterised by the continuous succession of flooding and drying periods and conditioned by many other farming activities (e.g. herbicide and pesticides spraying, grass mowing, rice harvesting etc.). Consequently, the orthopteran fauna in rice crops is mainly composed of species well adapted to sudden environmental changes, i.e. able to rapidly respond to disturbance events by relocating to new suitable habitats. On the contrary, sedentary species are often severely affected by habitat loss and detrimental human activities (Ribera et al. 2001), thus justifying their limited occurrence in a heavily managed ecosystem as the rice fields of western Lomellina.

In this context, intensive agriculture is likely to further restrict the occurrence of species with low mobility, therefore emerging as an important factor in conditioning orthopteran diversity in rice crops. In particular, the widespread reduction of semi-natural habitats may prevent the availability of stable ecosystems, often essential for the conservation of many sedentary and sensitive species. Moreover, the intensification of grass management on rice field banks can also strongly affect orthopteran diversity (Giuliano et al., *under review*), especially by limiting the occurrence of species specifically adapted to live in habitats characterised by a well-developed vegetation structure, as already demonstrated for ground beetles in this agroecosystem (Cardarelli and Bogliani 2014).

Lastly, hygrophilous orthopterans are today seriously threatened in rice crops by the introduction of new rice cultivation methods, which often involve limited water inputs. Indeed, the spread of dry cultivated rice fields may reduce the suitability of this agroecosystem for species with high moisture requirements, thus contributing to further aggravate their conservation status in Europe.

## Conclusions

Rice crops proved to be an important ecosystem for orthopteran diversity in western Lomellina, especially providing a suitable habitat for several hygrophilous species. This result confirms the value of rice fields for biodiversity conservation, already recognised for many other taxonomic groups (Fasola and Ruiz 1996, Lawler 2001, Czech and Parsons 2002, Bambaradeniya et al. 2004, Duré et al. 2008, Lupi et al. 2013).

Besides, conservation efforts should be applied in rice agroecosystems to limit the impacts of intensive agriculture on orthopteran assemblages, especially through the introduction of sustainable farming strategies. First of all, farmers should be provided with incentives for preserving and restoring marginal semi-natural habitats (e.g. uncultivated grasslands, wetlands etc.), in order to improve landscape heterogeneity and to ensure the occurrence of permanent biotopes. These features are often essential for biodiversity conservation in farmlands (Benton et al. 2003, Tscharntke et al. 2005), also enhancing the habitat availability for species with limited dispersal capacity, which are otherwise unable to survive and reproduce in rice fields.

Secondly, grass management intensity on paddy banks should be reduced by rice growers. This goal could be easily achieved by leaving uncut levee portions, proved to be an effective solution to control the detrimental impact of mowing on orthopterans (Humbert et al. 2012), as well as on ground beetles (Cardarelli and Bogliani 2014). In addition, the preservation of well-structured vegetation portions on rice field banks may also improve the occurrence of species with specific habitat requirements, producing consistent benefits for the overall orthopteran diversity.

Although the integrated application of these management strategies may increase the suitability of intensive rice crops for orthopterans, further detrimental agronomic practices, like rice cultivation in dry soils, should be discouraged in future, in order to preserve the environmental value of rice agroecosystems for these insects.

## Figures and Tables

**Table 1.** List of the orthopteran species collected in the rice agroecosystem of western Lomellina. For each species, information is reported about the period of observation (Phe; months in roman numerals), the mobility class (Mob), the habitat specificity class (HS) and habitat of occurrence. Mobility classes are: 1 = Sedentary; 2 = Intermediate dispersers; 3 = Mobile species. Habitat specificity classes are: 1 = Generalists; 2 = Medium specialised species; 3 = Specialists (* indicates hygrophilous species). Nomenclature follows Cigliano et al. (2018).

**Species**	**Phe**	**Mob**	**HS**	**Habitats**			
				Paddies	Banks	Canals	Farm roads
*Phaneroptera nana* Fieber, 1853	VII	3	2		+		
Conocephalus (Anisoptera) fuscus (Fabricius, 1793)	VI-IX	2	3*		+	+	
*Ruspolia nitidula* (Scopoli, 1786)	VII-IX	3	3*		+	+	
*Tettigonia viridissima* (Linnaeus, 1758)	VI-VIII	3	1		+		
*Decticus albifrons* (Fabricius, 1775)	VIII-IX	2	2		+		
*Roeseliana azami* (Finot, 1892)	VI-VIII	1	2		+		
Pteronemobius (Pteronemobius) heydenii (Fischer, 1853)	V-IX	1	3*	+		+	
Gryllus (Gryllus) campestris Linnaeus, 1758	V	1	2		+		
*Eumodicogryllus bordigalensis* (Latreille, 1804)	V-IX	1	2	+	+	+	
*Gryllotalpa* sp.	V-IX	2	2		+	+	
*Paratettix meridionalis* (Rambur, 1838)	VII-IX	3	3*	+	+	+	
*Tetrix ceperoi* (Bolivar, 1887)	VII	3	3*	+	+	+	
*Calliptamus italicus* (Linnaeus, 1758)	VII-VIII	2	2				+
*Locusta migratoria* Fabricius, 1781	VI-IX	3	2	+	+		+
*Oedipoda caerulescens* (Linnaeus, 1758)	VIII	3	2				+
*Oedipoda germanica* (Latreille, 1804)	VIII	1	2				+
*Acrotylus patruelis* (Herrich-Schaeffer, 1838)	VIII	3	2				+
*Aiolopus strepens* (Latreille, 1804)	IX	3	2				+
*Mecostethus parapleurus* (Hagenbach, 1822)	VI-VIII	2	2		+	+	
*Paracinema tricolor* (Thunberg, 1815)	VII-IX	3	3*	+	+		
Omocestus (Omocestus) rufipes (Zetterstedt, 1821)	VI-IX	2	2				+
Chorthippus (Chorthippus) dorsatus (Zetterstedt, 1821)	VII-IX	3	1		+		
Chorthippus (Glyptobothrus) brunneus (Thunberg, 1815)	VI-IX	3	2		+		+
*Pseudochorthippus parallelus* (Zetterstedt, 1821)	VI-VIII	3	1		+		+
*Euchortippus declivus* (Brisout de Barneville, 1848)	VII-IX	3	2				+
